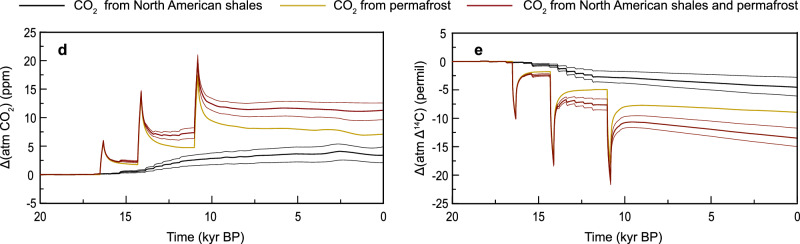# Author Correction: Deglacial release of petrogenic and permafrost carbon from the Canadian Arctic impacting the carbon cycle

**DOI:** 10.1038/s41467-025-59905-w

**Published:** 2025-05-27

**Authors:** Junjie Wu, Gesine Mollenhauer, Ruediger Stein, Peter Köhler, Jens Hefter, Kirsten Fahl, Hendrik Grotheer, Bingbing Wei, Seung-Il Nam

**Affiliations:** 1 Alfred-Wegener-Institut Helmholtz-Zentrum für Polar-und Meeresforschung (AWI), Bremerhaven, 27568 Germany; 2 MARUM–Center for Marine Environmental Sciences and Faculty of Geosciences, University of Bremen, Bremen, 28359 Germany; 3 Frontiers Science Center for Deep Ocean Multispheres and Earth System, and Key Laboratory of Marine Chemistry Theory and Technology, Ocean University of China, Qingdao, 266100 China; 4 State Key Laboratory of Marine Geology, Tongji University, Shanghai, 200092 China; 5 Korea Polar Research Institute, Incheon, 21990 Republic of Korea; 6 Present Address: Department of Environmental Science, Stockholm University, Stockholm, 11418 Sweden

Correction to: *Nature Communications* 10.1038/s41467-022-34725-4, published online 22 November 2022

In the version of the article initially published, due to an error in source files, the model presented in Fig. 5d, e overestimated emissions. In the Abstract, in the sentence now reading “Assuming extensive petrogenic organic carbon oxidation during the glacial retreat, a model-based assessment suggests that the combined processes have contributed 8 ppm to the deglacial CO_2_ rise,” 8 ppm replaces “12 ppm.” In the eleventh paragraph of the Discussion, values in the text have been updated to read “These events include a first CO_2_ peak of 3 ppm at 16.5 cal. kyr BP and two more CO_2_ peaks of ~6 ppm at 14.6 and 11.5 cal. kyr BP (Fig. 5d). These permafrost carbon release pulses lead in the model to a decrease in Δ14C of ~5 permil at 16.5 cal. kyr BP and of ~7–8 permil at 14.6 cal. kyr BP and 11.5 cal. kyr BP (Fig. 5e)….. The simulated long-term effects over the last 20 kyrs when combining both processes are an increase in atmospheric CO_2_ by 8 ppm and a decrease in atmospheric Δ14C by 9 permil (Figs. 5d, e), explaining 10% and 2%, respectively, of the reconstructed changes in both variables.” The paragraph previously read “These events include a first CO_2_ peak of 6 ppm at 16.5 cal. kyr BP and two more CO_2_ peaks of ~12 ppm at 14.6 and 11.5 cal. kyr BP (Fig. 5d). These permafrost carbon release pulses lead in the model to a decrease in Δ14C of ~10 permil at 16.5 cal. kyr BP and of ~13–15 permil at 14.6 cal. kyr BP and 11.5 cal. kyr BP (Fig. 5e)…. The simulated long-term effects over the last 20 kyrs when combining both processes are an increase in atmospheric CO_2_ by 12 ppm and a decrease in atmospheric Δ14C by 12 permil (Figs. 5d, e), explaining 13% and 3%, respectively, of the reconstructed changes in both variables.”

Panels in Fig. 5d, e have now been replaced; for comparison, the original Fig. 5d, e panels are shown as Fig. 1, below. The changes to figures and text are made in the HTML and PDF versions of the article.

Fig. 1 Original Fig. 5d, e.