# Supplementary material for: Deglacial release of petrogenic and permafrost carbon from the Canadian Arctic impacting the carbon cycle
Source: Nat Commun. 2022 Nov 22;13:7172. doi: 10.1038/s41467-022-34725-4 (PMC9684420; doi:10.1038/s41467-022-34725-4)
Supplement: Supplementary file 1 — Supplementary Information [file 41467_2022_34725_MOESM1_ESM.pdf]

Supplementary Information for

Deglacial release of petrogenic and permafrost carbon from the Canadian Arctic impacting the carbon cycle

Junjie Wu*, Gesine Mollenhauer*, Ruediger Stein*, Peter Köhler, Jens Hefter, Kirsten Fahl, Hendrik Grotheer, Bingbing Wei, Seung-Il Nam

*Corresponding author: junjie.wu@awi.de (J.W.); gesine.mollenhauer@awi.de (G.M.); rstein@marum.de (R.S.)

This PDF file includes:

Supplementary Discussion Texts 1 and 2

Supplementary Figures 1 to 8

Supplementary Tables 1 and 2

Supplementary Discussion

Text 1: Mixing model and endmember values

Mixing model

A three-endmember mixing model based on $\delta^{13}C_{org}$ and $F^{14}C$ signature was used to estimate fractional contributions of $OC_{marine\text{-}bio}$ ($f_{marine\text{-}bio}$), $OC_{terr\text{-}bio}$ ($f_{terr\text{-}bio}$), and OC_{petro} (f_{petro}), assuming mass-balance:

$$F^{14}C_{bulk\text{-}ini} = f_{marine\text{-}bio} \times F^{14}C_{marine\text{-}bio} + f_{terr\text{-}bio} \times F^{14}C_{terr\text{-}bio} + f_{petro} \times F^{14}C_{petro} \quad (1)$$

$$\delta^{13}C_{bulk} = f_{marine\text{-}bio} \times \delta^{13}C_{marine\text{-}bio} + f_{terr\text{-}bio} \times \delta^{13}C_{terr\text{-}bio} + f_{petro} \times \delta^{13}C_{petro} \quad (2)$$

$$1 = f_{marine\text{-}bio} + f_{terr\text{-}bio} + f_{petro} \quad (3)$$

The $\delta^{13}C_{bulk}$ and $F^{14}C_{bulk\text{-}ini}$ denote the values of analyzed bulk sediments. In this case, the $F^{14}C_{bulk\text{-}ini}$ refers to fraction modern carbon before deposition ($F^{14}C_{initial} = F^{14}C \times e^{\lambda t}$, t is the time since deposition). The $\delta^{13}C_{bulk}$ is directly measured in sediment samples. Other isotope values are source characteristics (the definition of each endmember value is explained below).

To obtain correct estimates, a Markov chain Monte Carlo Bayesian approach (MCMC) was applied to include uncertainty of source-signature $\delta^{13}C$ and $F^{14}C$, minimizing errors from arbitrary assignments of endmember values. Analytical errors of the samples are not included in the mixing model, as they are considered negligible compared to the endmember uncertainties. The simulation was performed on MATLAB, based on the script from Andersson et al. (2015)[1]. Briefly, 1,000,000 out of 100,000,000 random data from the normal distribution of each endmember within the given mean and standard deviation were taken to simultaneously fulfill the given system in simulations. The mean relative contributions and the standard deviation of different OC pools were then estimated.

Endmembers

Marine biospheric carbon

Since no proper $\delta^{13}C$ and $F^{14}C$ data from local phytoplankton are available, $\delta^{13}C_{marine\text{-}bio}$ is defined as −24.0±1.0‰, using data from the Siberian Arctic shelf[2]. $F^{14}C_{marine\text{-}bio}$ largely depends on the radiocarbon content of dissolved inorganic carbon (DIC) in surface waters.

Thus, $F^{14}C_{marine\text{-}bio}$ has been assumed the same as $F^{14}C_{surface}$ (fraction modern carbon of DIC in surface waters). The marine reservoir age R indicates the offset metric between the ^{14}C contents of reservoirs of the ocean surface and the atmosphere, which can be expressed as the Supplementary Eq.4[3]:

$$R = -8033 \times \ln \frac{F^{14}C_{surface}}{F^{14}C_{atmosphere}} \quad (4)$$

Hence, $F^{14}C_{surface}$ ($F^{14}C_{marine\text{-}bio}$) has been derived based on the $F^{14}C_{atmosphere}$ from IntCal13[4] and the regional marine reservoir age R. Note that $F^{14}C_{atmosphere}$ varies through time, and thus a variable $F^{14}C_{marine\text{-}bio}$ is used in the mixing model. R in high latitudes might be larger than the global average (405 years) due to the sea ice impact and is simulated to be 1000 years in the Canadian Beaufort Sea[5]. In the nearby regions of the western Arctic Ocean, ΔR has been determined to be 440±101 years in the Chukchi/Beaufort, 350±116 years in the Amundsen Gulf, and 286±74 years in the Foxe Basin (cf., ref[6]). Collectively, the regional R in the Canadian Beaufort Sea appears to range between 405-1000 years. In order to validate the robustness of our mixing model, both $F^{14}C_{marine\text{-}bio}$ values derived from scenarios of R=405 years and R=1000 years are tested in the model (see below). Additionally, due to the ocean circulation change, the sea surface reservoir ages were 200 years greater during the YD[7]. Thus, the regional R=605 and R=1200 are used for the YD period. Figure 4b shows the variable $F^{14}C_{marine\text{-}bio}$ that we used in the mixing model (based on the scenario of R=405 years).

Uncertainties (σ) on reservoir age offset R are functions of the $F^{14}C$ values of two reservoirs and their associated uncertainties[3]:

$$\sigma_R = 8033 \times \sqrt{\left(\frac{\sigma_{surface}}{F^{14}C_{surface}}\right)^2 + \left(\frac{\sigma_{atmosphere}}{F^{14}C_{atmosphere}}\right)^2} \quad (5)$$

Here we assume an uncertainty of 100 years for the reservoir age R (to keep consistency with the uncertainties used in the age model) and derive the uncertainties of $F^{14}C_{surface}$ ($F^{14}C_{marine\text{-}bio}$).

Terrestrial biospheric carbon

The majority of terrestrial OC delivered to the study site is discharged from the Mackenzie River. In this study, source characteristics from Herschel Island and Yukon Coast have been considered as well since these regions are characterized by high erosion rates and may contribute to significant terrestrial carbon release. A study of soil organic carbon on a north-south transect in western Canada (including the Mackenzie River basin) suggests a mean $\delta^{13}C_{terr\text{-}bio}$ value of −26.2±0.5‰[8]. Analysis of samples from the Herschel Island retrogressive thaw slumps (n=7) and onshore samples from the Yukon Coastal Plain (n=19) gives a mean $\delta^{13}C_{terr\text{-}bio}$ value of −26.5±0.3‰[9,10], which is within the range reported by Bird et al. (2002)[8]. Therefore, the endmember $\delta^{13}C$ value of terrestrial biospheric carbon is defined as −26.2±0.5‰ in our mixing model.

Defining the endmember $F^{14}C$ value of terrestrial biospheric carbon needs to consider the dynamics of ice sheets, permafrost formation, and vegetation development during the ice sheet retreat. In this context, the relatively fixed endmember values from the contemporary system are not representative of these changes and thus might not be applicable to the paleo system. Instead, HMW n-alkanoic acids derived from the study material can to some degree reflect the dynamic changes for different periods. Besides, using HMW n-alkanoic acids relieves the constraint of finding proper endmember values to represent all possible sources. One should also consider that the endmember values determined on bulk OC on land are fixed while the radiocarbon signals of terrestrial OC delivered to the study site may change (e.g., become depleted) via preferential degradation or re-suspension during transport, and such biases may eventually cause an underestimate of $OC_{terr\text{-}bio}$ contribution. In contrast, using HMW n-alkanoic acids values determined from the study site circumvents these additional complications. Hence, we define the $F^{14}C$ of HMW-FAs (corrected for depositional decay) as endmember values of terrestrial biospheric carbon.

Petrogenic organic carbon

OC_{petro} is radiocarbon free and the endmember $F^{14}C_{petro}$ is defined as 0. Unfortunately, studies on the stable carbon isotopic composition of OC_{petro} are scarce in the Mackenzie River basin. Hence, we turn to the nearby regions to obtain representative endmember $\delta^{13}C$ values.

There are three geological units in the Mackenzie River catchment: North American Cordillera, the Interior Platform, and the Canadian Shield. The North American Cordillera and central Interior Platform are likely the major contributors of OC_{petro}[11]. According to the geological map

of Canada, the two geological units in the catchment mainly include Phanerozoic sedimentary rocks, more specifically Cambrian to Cretaceous strata. Records from Alberta[12] can serve to provide information on the characteristics of sedimentary rocks from the Interior Platform, while records from British Columbia[13,14] can provide constraints on sedimentary rocks from the Cordillera. These records have further been placed in a global compilation and are consistent with the $\delta^{13}C$ values of kerogens from various Phanerozoic sedimentary rocks[15] (Supplementary Fig. 3).

The $\delta^{13}C$ data from Canadian sedimentary rocks gives a mean value of −29.0±1.4‰. However, these records mainly cover the Permian to Jurassic. Therefore, a mean $\delta^{13}C$ value of −28.6±1.3‰ of Cambrian to Cretaceous rocks has been calculated from a global compilation[15]. Both $\delta^{13}C$ values have been used to test the robustness of the mixing model output (see below).

Robustness of mixing model

To test the model sensitivity, endmember $F^{14}C_{marine\text{-}bio}$ values derived from scenarios of R=405 years and R=1000 years are tested respectively. Endmember values of each scenario are defined as follows:

Scenario 1: $F^{14}C_{marine\text{-}bio}$ values are derived from the scenario of R=405 years, and the $\delta^{13}C_{petro}$ value is defined as −28.6±1.3‰. Please see the definitions of other endmember values above.

Scenario 2: $F^{14}C_{marine\text{-}bio}$ values are derived from the scenario of R=1000 years, and the $\delta^{13}C_{petro}$ value is defined as −28.6±1.3‰. Please see the definition of other endmember values above.

Under both circumstances, the outcome remains the same (Supplementary Figure 4), indicating that our results are robust within the possible range of R. Therefore, we use $F^{14}C_{marine\text{-}bio}$ values derived from the scenario of R=405 years for the following tests.

The $\delta^{13}C_{petro}$ data from both British Columbia and Alberta gives a mean $\delta^{13}C_{petro}$ value of −29.0±1.4‰ (n=436), which has been tested in Scenario 3.

Scenario 3: $F^{14}C_{marine\text{-}bio}$ values are derived from the scenario of R=405 years, and the $\delta^{13}C_{petro}$ value is defined as −29.0±1.4‰. Please see the definitions of other endmember values above.

The model outcome shows negligible differences with using the $\delta^{13}C_{petro}$ value from the global compilation (Supplementary Figure 4), suggesting that the results are robust within the possible $\delta^{13}C_{petro}$ range.

Because of the less constrained chronology at the top and the base of the core, uncertainties of depositional age should be taken into consideration which may impact the sample $F^{14}C_{bulk-ini}$ and endmember $F^{14}C_{terr-bio}$ and may further influence the mixing model output. Note that age biases impact both $F^{14}C_{bulk-ini}$ and $F^{14}C_{terr-bio}$, hence uncertainties cannot be simply assigned to the sample $F^{14}C_{bulk-ini}$ and endmember $F^{14}C_{terr-bio}$. Instead, we incorporate maximum and minimum depositional ages in the calculation respectively, as indicated in Scenarios 4 and 5.

Scenario 4: Maximum depositional ages are incorporated in the calculation of $F^{14}C_{bulk-ini}$ and $F^{14}C_{terr-bio}$. $F^{14}C_{marine-bio}$ values are derived from the scenario of R=405 years, and the $\delta^{13}C_{petro}$ value is defined as −28.6±1.3‰. Please see the definitions of other endmember values above.

Scenario 5: Minimum depositional ages are incorporated in the calculation of $F^{14}C_{bulk-ini}$ and $F^{14}C_{terr-bio}$. $F^{14}C_{marine-bio}$ values are derived from the scenario of R=405 years, and the $\delta^{13}C_{petro}$ value is defined as −28.6±1.3‰. Please see the definitions of other endmember values above.

The outcome of Scenarios 4 and 5 illustrates a negligible influence of age model uncertainties (Supplementary Figure 4).

Overall, the exercises demonstrate a robust mixing model outcome. Different scenarios have negligible influence on our estimate. As the mixing model output remains nearly the same in all scenarios, we discuss in the Main Text based on the mixing model outcome of Scenario 1 which is shown in Supplementary Table 1.

Text 2: Mass accumulation rates of organic carbon

Changes in OC_{petro} MARs are used in the Main Text to reflect changes in OC_{petro} oxidation flux. Since our mixing model provides a robust outcome of source fractions of OC_{petro}, defining TOC MARs becomes crucial for estimating OC_{petro} MARs. TOC MARs show three distinct peaks between 14-10 cal. kyr BP (Fig. 3d), triggered by the YD flood event and two more events of coastal erosion. In addition to the influence of these strong events, the sharp increase in TOC MARs may be partially driven by the limited dating points in the age model. It implies that using TOC MARs from these intervals may cause an overestimate of OC_{petro} MARs.

In this context, for samples deposited between 10-0 cal. kyr BP, the samples' respective TOC MARs are used to calculate OC_{petro} MARs. However, for samples deposited between 14-10 cal. kyr BP, we tested three scenarios using different TOC MARs. In a conservative estimate, the

average value of low TOC MARs (values in between events) between 14-10 cal. kyr BP is used to estimate OC_{petro} MARs (Supplementary Figure 5a). In the second scenario, the average TOC MAR derived from the entire period of 14-10 cal. kyr BP is used (Supplementary Figure 5b). In scenario 3, the samples' respective TOC MARs are used (Supplementary Figure 5c).

The OC_{petro} MARs discussed in the Main Text are calculated from a conservative estimate of TOC MARs.

Supplementary Figures

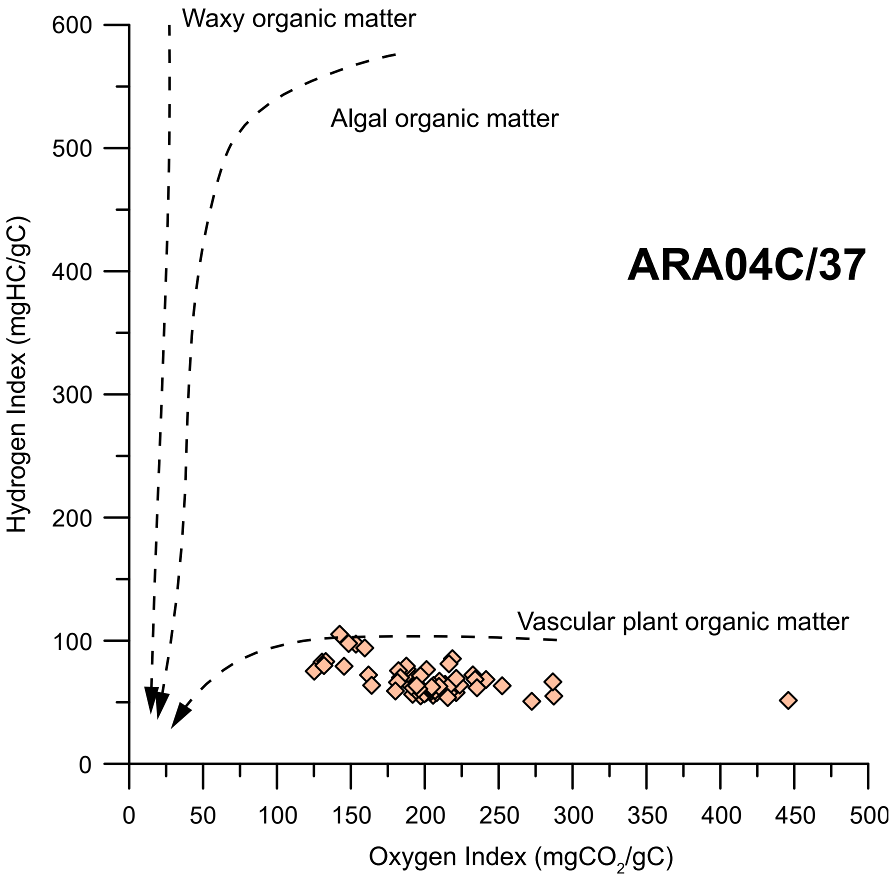

Supplementary Figure 1. Carbon sources based on pyrolysis. The pseudo van Krevelen-type diagram shows hydrogen index and oxygen index for bulk OC. The dashed lines illustrate different carbon sources (cf., ref[16]).

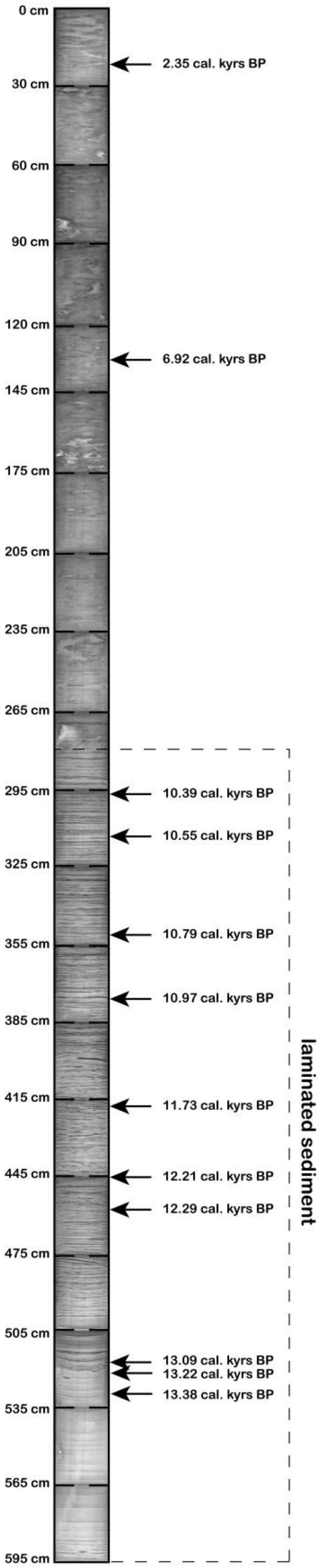

Supplementary Figure 2. **Lithology of core ARA04C/37.** X-ray digital radiograph showing lithology and sedimentary texture (bioturbation and lamination) of core ARA04C/37.

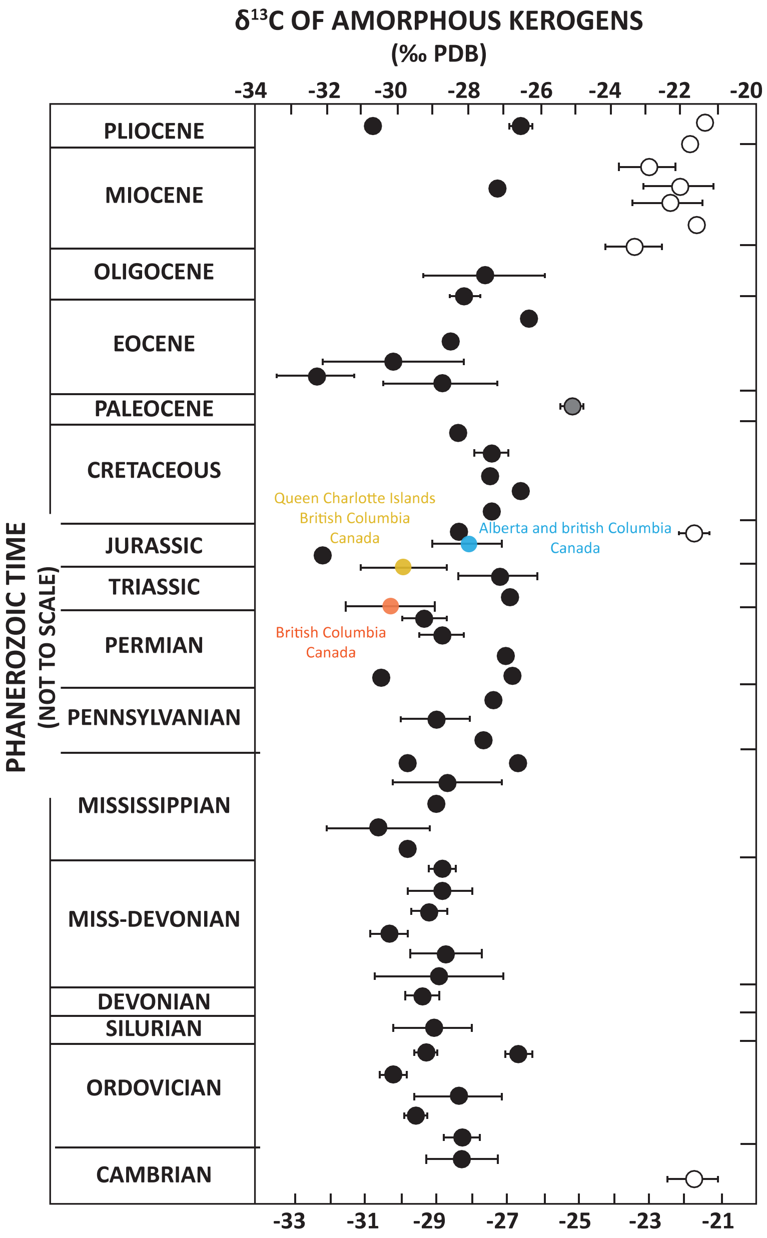

Supplementary Figure 3. stable carbon isotopes of kerogens from Phanerozoic rock units. Colour circles indicate the stable isotope composition of rocks from Canada[12–14]. White circles denote *h*-amorphous kerogens, and black circles denote *l*-amorphous kerogens from various regions[15]. Error bars represent standard deviation.

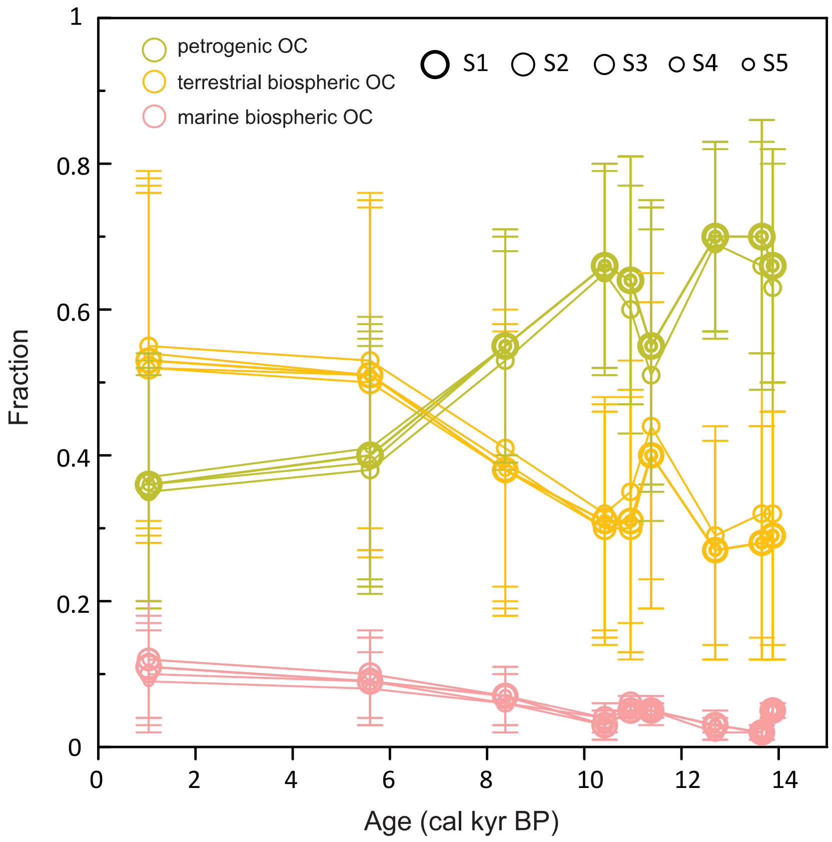

Supplementary Figure 4. **Mixing model sensitivity.** The plots show the results of source apportionments for samples in core ARA04C/37. Different sizes of circles indicate the results of scenarios 1-5 (S1-S5) discussed in Supplementary Discussion Text 1. Error bars represent standard deviation.

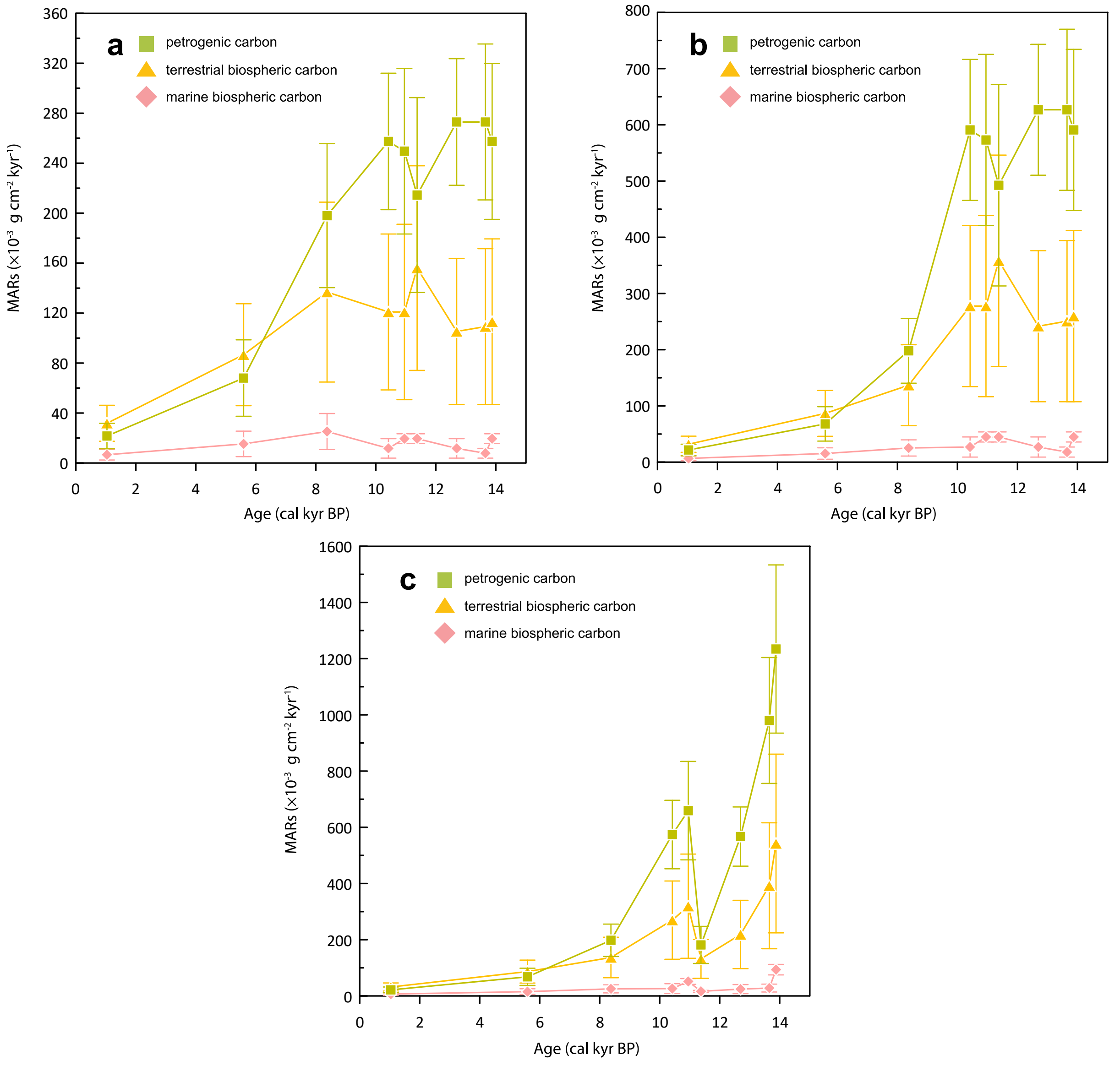

Supplementary Figure 5. **Mass accumulation rates (MARs) of different types of carbon. a-c** show the MARs of OC_{petro}, $OC_{terr\text{-}bio}$, and $OC_{marine\text{-}bio}$. In **a** and **b**, MARs between 14-10 cal. kyr BP are calculated based on an average value of low TOC MARs (values in between events of high MAR – see Figure 3d) (**a**) and an average TOC MAR from the entire period of 14-10 cal. kyr BP (**b**), while in panel **c** MARs are calculated based on samples' respective TOC MARs (Supplementary Discussion Text 2). Error bars represent standard deviation.

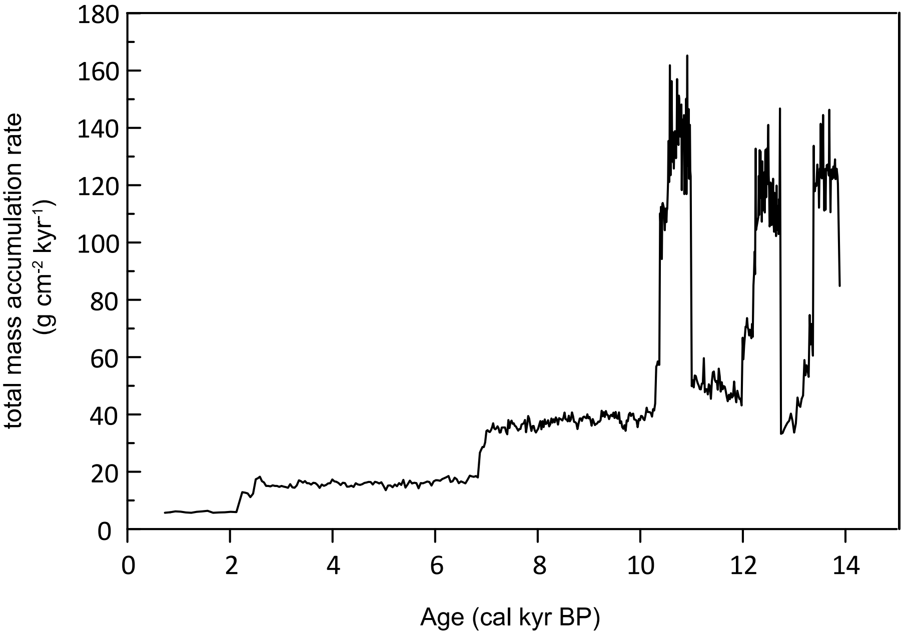

Supplementary Figure 6. **Total mass accumulation rates of ARA04C/37.** The total mass accumulation rates are updated from Wu et al. (2020)[17] by including two more AMS^{14}C dates (Supplementary Table 2).

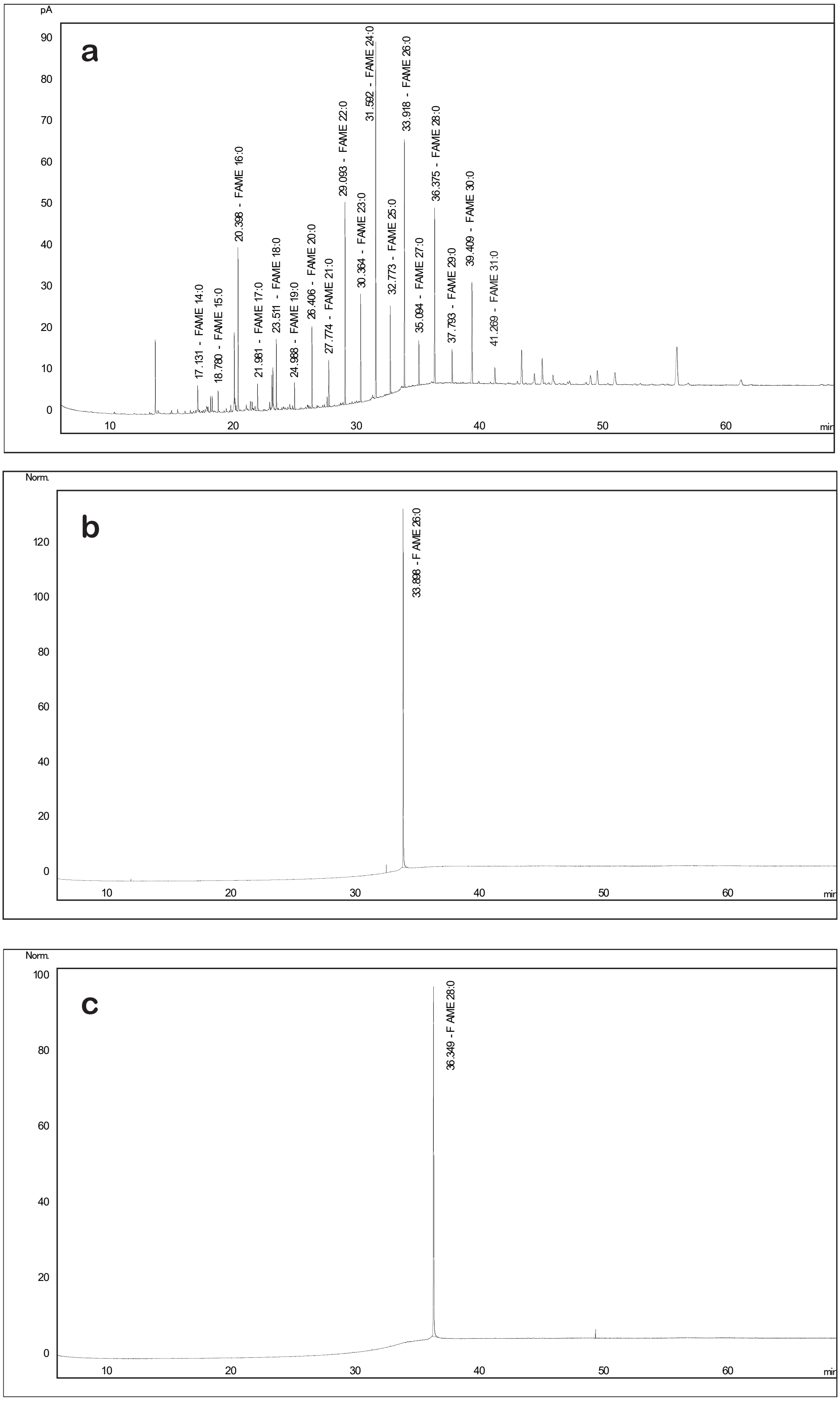

Supplementary Figure 7. **Representative chromatograms of *n*-alkanoic acids in core ARA04C/37. a** representative chromatogram of *n*-alkanoic acids. **b-c** representative chromatograms of $C_{26:0}$ and $C_{28:0}$ fatty acids that are used for compound-specific radiocarbon analyses.

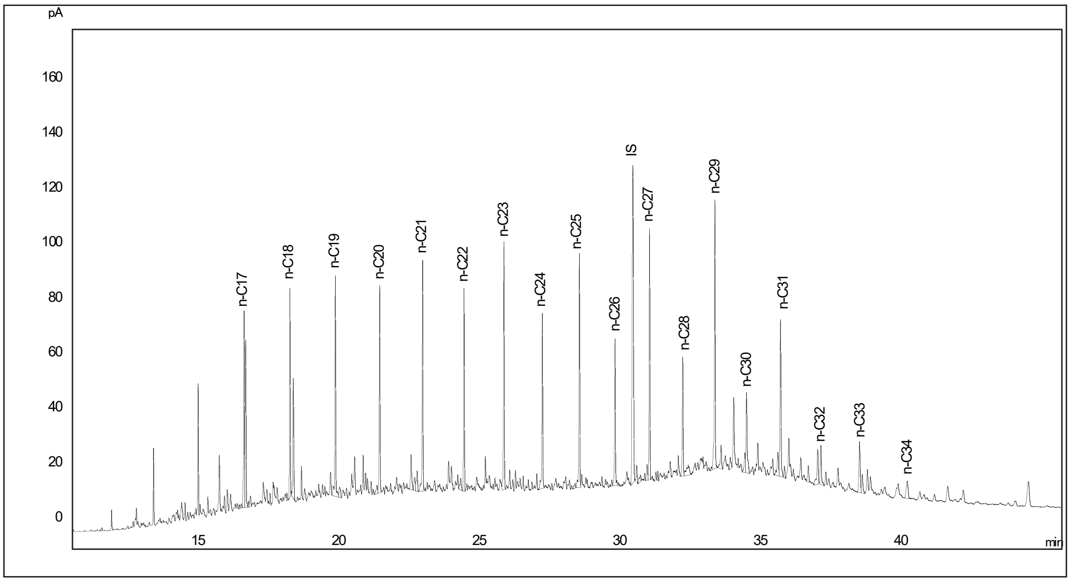

Supplementary Figure 8. **Representative chromatogram of an *n*-alkanes fraction extracted from sediments of core ARA04C/37.** IS denotes the internal standard squalane.

Supplementary Tables

Supplementary Table 1. Source fractions of marine biospheric carbon, terrestrial biospheric carbon, and petrogenic organic carbon, based on a three-endmember mixing model.

Depth (cm)	Age (cal. kyr BP)	Marine biospheric carbon (mean±s.d.)	Terrestrial biospheric carbon (mean±s.d.)	Petrogenic organic carbon (mean±s.d.)
9	1.036	0.11±0.07	0.53±0.24	0.36±0.17
99	5.592	0.09±0.06	0.51±0.24	0.40±0.18
199	8.374	0.07±0.04	0.38±0.20	0.55±0.16
299	10.420	0.03±0.02	0.31±0.16	0.66±0.14
372	10.952	0.05±0.01	0.31±0.18	0.64±0.17
399	11.376	0.05±0.01	0.40±0.21	0.55±0.20
499.5	12.695	0.03±0.02	0.27±0.15	0.70±0.13
564	13.653	0.02±0.01	0.28±0.16	0.70±0.16
593	13.877	0.05±0.01	0.29±0.17	0.66±0.16

Supplementary Table 2. AMS^{14}C dates of core ARA04C/37

Lab ID	Depth (cm)	Species	AMS^{14}C age (year)	△R	Calibrated age* (cal. year BP)	Model age** (cal. year BP)
6472.1.2	521	planktic	11756±101	0±100	13233±140	13220±123
6474.1.2	530	planktic	12095±104	0±100	13553±154	13381±157

AMS^{14}C dating of the core ARA04C/37 has been carried out at the Alfred Wegener Institute Bremerhaven using the Mini Carbon Dating System (MICADAS)[18].

*Calibrated ages with 1σ uncertainty, using Calib 7.1[19] and based on calibration curve Marine13[4].

**model ages with default 95% confidence ranges based on Bayesian model (Bacon)[20] and Marine13[4] in this study.

Supplementary References

1. Andersson, A. *et al.* Regionally-Varying Combustion Sources of the January 2013 Severe Haze Events over Eastern China. *environmental science & Technology* **49**, 2038–2043 (2015).

2. Vonk, J. E. *et al.* Preferential burial of permafrost-derived organic carbon in Siberian-Arctic shelf waters. *Journal of Geophysical Research: Oceans* **119**, 8410–8421 (2014).

3. Soulet, G., Skinner, L. C., Beaupré, S. R. & Galy, V. A note on reporting of reservoir 14C disequilibria and age offsets. *Radiocarbon* **58**, 205–211 (2016).

4. Reimer, P. J. *et al.* Intcal13 and Marine13 Radiocarbon Age Calibration Curves 0–50,000 Years Cal Bp. *Radiocarbon* **55**, 1869–1887 (2013).

5. Butzin, M., Köhler, P. & Lohmann, G. Marine radiocarbon reservoir age simulations for the past 50,000 years. *Geophysical Research Letters* **44**, 8473–8480 (2017).

6. Keigwin, L. D. *et al.* Deglacial floods in the Beaufort Sea preceded Younger Dryas cooling. *Nature Geoscience* **11**, 599–604 (2018).

7. Bondevik, S., Mangerud, J., Birks, H. H., Gulliksen, S. & Reimer, P. Changes in North Atlantic Radiocarbon Reservoir Ages During the Allerød and Younger Dryas. *Science* **312**, 1514–1517 (2006).

8. Bird, M., Santrùcková, H., Lloyd, J. & Lawson, E. The isotopic composition of soil organic carbon on a north-south transect in western Canada. *European Journal of Soil Science* **53**, 393–403 (2002).

9. Grotheer, H. *et al.* Burial and origin of permafrost derived carbon in the nearshore zone of the southern Canadian Beaufort Sea. *Geophysical Research Letters* **47**, e2019GL085897 (2020).

10. Couture, N. J., Irrgang, A., Pollard, W., Lantuit, H. & Fritz, M. Coastal Erosion of Permafrost Soils Along the Yukon Coastal Plain and Fluxes of Organic Carbon to the Canadian Beaufort Sea. *Journal of Geophysical Research: Biogeosciences* **123**, 406–422 (2018).

11. Campeau, A., Soerensen, A., Martma, T., Åkerblom, S. & Zdanowicz, C. Controls on the 14 C-content of dissolved and particulate organic carbon mobilized across the Mackenzie River basin, Canada. *Global Biogeochemical Cycles* **34**, e2020GB006671 (2020).

12. Them, T. R. *et al.* High-resolution carbon isotope records of the Toarcian Oceanic Anoxic Event (Early Jurassic) from North America and implications for the global drivers of the Toarcian carbon cycle. *Earth and Planetary Science Letters* **459**, 118–126 (2017).

13. Wang, K., Geldsetzer, H. H. J. & Krouse, H. R. Permian-Triassic extinction: Organic δ13 C evidence from British Columbia, Canada. *Geology* **22**, 580–584 (1994).

14. Williford, K. H., Ward, P. D., Garrison, G. H. & Buick, R. An extended organic carbon-isotope record across the Triassic-Jurassic boundary in the Queen Charlotte Islands, British Columbia, Canada. *Palaeogeography, Palaeoclimatology, Palaeoecology* **244**, 290–296 (2007).

15. Lewan, M. D. Stable carbon isotopes of amorphous kerogens from Phanerozoic sedimentary rocks. *Geochimica et Cosmochimica Acta* **50**, 1583–1591 (1986).

16. Tissot, B. P. & Welte, D. H. *Petroleum Formation and Occurrence*. (Springer-Verlag, 1984).

17. Wu, J. *et al.* Deglacial to Holocene variability in surface water characteristics and major fl oods in the Beaufort Sea. *Communications Earth & Environment* **1**, 27 (2020).

18. Mollenhauer, G., Grotheer, H., Gentz, T., Bonk, E. & Hefter, J. Standard operation procedures and performance of the MICADAS radiocarbon laboratory at Alfred Wegener Institute (AWI), Germany. *Nuclear Instruments and Methods in Physics Research, Section B: Beam Interactions with Materials and Atoms* **496**, 45–51 (2021).

19. Stuiver, M., Reimer, P. J. & Reimer, R. W. CALIB 7.1 [WWW program] at http://calib.org. (2020).

20. Blaauw, M. & Christeny, J. A. Flexible paleoclimate age-depth models using an autoregressive gamma process. *Bayesian Analysis* **6**, 457–474 (2011).
